# Segregation of Seizure Traits in C57 Black Mouse Substrains Using the Repeated-Flurothyl Model

**DOI:** 10.1371/journal.pone.0090506

**Published:** 2014-03-03

**Authors:** Sridhar B. Kadiyala, Dominick Papandrea, Bruce J. Herron, Russell J. Ferland

**Affiliations:** 1 Center for Neuropharmacology and Neuroscience, Albany Medical College, Albany, New York, United States of America; 2 Wadsworth Center, Albany, New York, United States of America; 3 Department of Biomedical Sciences, School of Public Health, University at Albany - State University of New York, Albany, New York, United States of America; 4 Department of Neurology, Albany Medical College, Albany, New York, United States of America; University of Modena and Reggio Emilia, Italy

## Abstract

Identifying the genetic basis of epilepsy in humans is difficult due to its complexity, thereby underlying the need for preclinical models with specific aspects of seizure susceptibility that are tractable to genetic analyses. In the repeated-flurothyl model, mice are given 8 flurothyl-induced seizures, once per day (the induction phase), followed by a 28-day rest period (incubation phase) and final flurothyl challenge. This paradigm allows for the tracking of multiple phenotypes including: initial generalized seizure threshold, decreases in generalized seizure threshold with repeated flurothyl exposures, and changes in the complexity of seizures over time. Given the responses we previously reported in C57BL/6J mice, we analyzed substrains of the C57BL lineage to determine if any of these phenotypes segregated in these substrains. We found that the generalized seizure thresholds of C57BL/10SNJ and C57BL/10J mice were similar to C57BL/6J mice, whereas C57BL/6NJ and C57BLKS/J mice showed lower generalized seizure thresholds. In addition, C57BL/6J mice had the largest decreases in generalized seizure thresholds over the induction phase, while the other substrains were less pronounced. Notably, we observed only clonic seizures during the induction phase in all substrains, but when rechallenged with flurothyl after a 28-day incubation phase, ∼80% of C57BL/6J and 25% of C57BL/10SNJ and C57BL/10J mice expressed more complex seizures with tonic manifestations with none of the C57BL/6NJ and C57BLKS/J mice having complex seizures with tonic manifestations. These data indicate that while closely related, the C57BL lineage has significant diversity in aspects of epilepsy that are genetically controlled. Such differences further highlight the importance of genetic background in assessing the effects of targeted deletions of genes in preclinical epilepsy models.

## Introduction

While mapping seizure-related quantitative trait loci (QTL) and identifying genes responsible for modifying baseline seizure threshold has been successful in rodents [1]–[9], discovery of genes beyond initial seizure threshold (e.g., changes in seizure threshold over time, development of more complex seizures, and/or epileptogenesis) remains challenging. This is in part due to a limited number of preclinical models for studying such complex traits. However, identifying genes beyond baseline seizure threshold is critical, since they could be targeted for therapeutic intervention possibly leading to better treatments for epilepsy. To this end, we have employed a mouse model of epilepsy using repeated exposure to the chemoconvulsant flurothyl, which we refer to as the repeated-flurothyl model, and have utilized this approach to investigate the genetic and environmental factors that influence seizure progression and seizure complexity [10]–[12].

While there are similarities between the repeated-flurothyl model and electrical or chemical kindling [13], we believe that the repeated-flurothyl model has several advantages over these seizure paradigms. Unlike the electrical kindling model, there is no need for the implantation of electrodes, thereby making the repeated-flurothyl model higher throughput. Unlike traditional chemical kindling models, flurothyl is inhaled; therefore there is less experimental error due to injection variability issues. Importantly, flurothyl seizures can be induced repeatedly without toxicity or ill effects [12]. If the administration of flurothyl continues for a sufficient period of time, seizures always occur. Therefore, the latency to the onset of flurothyl seizures represents a direct measure of seizure susceptibility. The greatest advantage of the repeated-flurothyl model is that this paradigm results in a progression of seizure behaviors that begin as clonic seizures, but change over time to seizures with tonic manifestations. This change in seizure complexity, which involves the interaction of two independent seizure expression networks (the forebrain and brainstem seizure networks [12], [14]–[21]), is not observed in other kindling models. Consequently, this alteration in seizure phenotype allows for the dissection of genes and mechanisms that are responsible for the propagation of ictal discharge from the forebrain seizure circuitry mediating clonic seizure expression to the brainstem seizure circuitry that mediates tonic seizure expression. This aspect of the repeated-flurothyl model is unique in that it provides a framework for better understanding why humans with epilepsy can develop more complex seizures over time [22]–[25]. Lastly, previous work has identified critical subcortical structures that are involved in mediating this change in seizure phenotype, further allowing for the mechanistic dissection of molecular processes responsible for this reorganization in mice exposed to the repeated-flurothyl model [26]. The recent elucidation of the importance of subcortical structures in the expression of generalized seizures in the human epileptic population particularly supports the importance of understanding the molecular processes controlling this phenotype in this preclinical model [27], [28].

Previously, we determined that C57BL/6J (6J) and DBA/2J (D2) mice have divergent seizure responses following exposure to the repeated-flurothyl model [11], [29]. Whereas 6J mice had higher initial generalized seizure thresholds (GST) with flurothyl, D2 mice had lower initial GST. Interestingly, 6J mice also had decreased GST following eight repeated flurothyl-induced seizures with D2 mice having no decreases in GST across these eight seizure trials [11]. Lastly, following these eight flurothyl seizure trials and a 28-day incubation period and final flurothyl rechallenge, 6J mice have a change in their seizure phenotype, which does not occur in D2 mice. This change in seizure phenotype occurred as a result of presumptive reorganizational (epileptogenic) changes in the brain, such that when 6J mice were retested with flurothyl, they developed more complex seizures (clonic seizures that uninterruptedly progressed into brainstem seizures)[11], [29]. Thus, elucidating the mechanisms that cause these differences in seizure responsivity between 6J and D2 mice can lead to the discovery of genes that can modify these seizure traits. Such information is critical for understanding these mechanisms and for developing strategies to target these processes.

Since the genetic diversity between 6J and D2 mice is comparatively high, in relation to the C57BL substrains, it is difficult to determine if these sub-phenotypes are due to a small number of QTLs affecting all of the traits, or if some or all of these traits can act independently. Previous work has shown seizure susceptibility differences to pilocarpine in two C57BL substrains, C57BL6 mice and C57BL6/N mice, and found that even within these substrains, the choice of animal vendor can affect seizure susceptibility (presumably through environmental factors or genetic drift) [30], [31]. Thus, our goal in this work was to investigate seizure behaviors in more divergent substrains of the C57BL line to determine if they were similar or divergent in various aspects of seizure progression.

## Materials and Methods

### Ethics Statement

All testing was performed under approval of the Institutional Animal Care and Use Committees of the Albany Medical College in accordance with The National Institutes of Health's *Guide for the Care and Use of Laboratory Animals*.

### Animals

Adult male C57BL/6J (6J; n = 12); C57BL/10SNJ (10SNJ; n = 12); C57BL/10J (10J; n = 12); C57BL/6NJ (6NJ; n = 12); C57BLKS/J (KSJ; n = 12) mice were obtained from Jackson Laboratories at 6 weeks of age (Bar Harbor, ME, USA). Mice were allowed to acclimate to the animal facility for one week before seizure testing commenced. Mice were housed on 12 hour light-dark cycle with ad libitum access to food and water.

### Experimental Design

Mice were exposed to the repeated-flurothyl model as previously described [10]–[12], [26], [29], [32]–[37]. Briefly, mice were placed in a closed chamber and a 10% flurothyl solution (bis(2,2,2-trifluoroethyl) ether; Sigma-Aldrich) made in 95% ethanol was infused through a glass syringe on to a gauze pad suspended at the top of the chamber at a rate of 100 µl/min using a motorized syringe pump. One mouse at a time was tested in the flurothyl chamber using a new gauze pad for each trial. The latency to the first myoclonic jerk expressed before the onset of a generalized seizure was recorded. Myoclonic jerks were defined by brief, but severe, contractions of the neck and body musculature occurring while the mouse maintained postural control [10], [12], [29]. The latency from the start of the flurothyl infusion to the expression of a myoclonic jerk was used as a measurement of the myoclonic jerk threshold (MJT)[29]. A generalized seizure was defined as a loss of postural control [10], [12]. When a mouse had a generalized seizure, the top of the chamber was removed exposing the mouse to room air. The latency from the start of the flurothyl infusion to the loss of postural control was used as a measurement of the generalized seizure threshold (GST)[10], [12]. Mice received a single flurothyl-induced seizure each day for 8 consecutive days (induction phase). The induction phase was followed by a 28-day incubation phase in which the mice were simply placed in the animal facility. After the incubation phase, mice were rechallenged with flurothyl.

The seizure behaviors were scored according to a number classification system [10], [12]: Grade 1 – a loss of posture, clonus of hindlimbs and/or forelimbs, and facial clonus including chewing; Grade 2 -grade 1 and low intensity bouncing; Grade 3 - grade 2 and wild running and hopping; Grade 4 - grade 3 and hindlimb and/or forelimb treading; Grade 5 - grade 4 and bilateral tonic extension of the forelimbs; Grade 6 - grade 5 and bilateral extension of the hindlimbs; and Grade 7 - grade 6 followed by death. Importantly, seizure grades 1–2 are classified as forebrain seizures, since these seizures are clonic in nature and involve forebrain structures for their expression [12], [38]–[42]. Seizure grades 3–7 denote a seizure type that begins as a clonic (forebrain) seizure where the animal losses postural control, regains posture, and rapidly progresses to a seizure with tonic manifestations (brainstem seizure). Therefore, we refer to such seizures as forebrain→brainstem seizures. Such seizures are denoted brainstem, since their seizure expression is controlled by a brainstem seizure network [17]–[19], [32], [37], [39], [43].

### Genetic and genomic analysis

Heritability (*H^2^*) was determined by dividing the between-strain variance by the sum of the within-strain and between-strain variance. Haplotype diversity was investigated for the three major substrains used in this study through publically available data and tools on the Mouse Phylogeny Viewer [44].

### Statistical analysis

One-way analysis of variance (ANOVA) followed by Newman-Keuls post-hoc comparisons were used to assess changes between strains. Repeated measures ANOVA followed by Newman-Keuls post-hoc comparisons were utilized to determine significance across repeated flurothyl seizure induction trials (kindling). GST and MJT on day 8 of the induction phase and on retest following incubation were compared using Student's t-test. Chi-square analysis was used to compare the percentage of animals changing their seizure phenotype. The point biserial correlation coefficient was used to determine correlation coefficients between the change in seizure phenotype and the other seizure parameters measured. Regression analysis was performed for parametric data. Statistical analyses were performed using Statistica (StatSoft).

## Results

Given the unique seizure characteristics of C57BL/6J (6J) mice [11], [12], [26], [32], [33], we examined substrains of C57BL mice to determine their initial myoclonic jerk threshold (MJT; as determined by the latency from the start of flurothyl infusion to the appearance of myoclonic jerks [12], [29]), decreases in MJT with repeated flurothyl exposures, initial generalized seizure threshold (GST; as determined by the latency from the start of flurothyl infusion to the expression of a generalized seizure [11], [12], [26], [32], [33]), decreases in GST with repeated flurothyl exposures, and the evolution of more complex seizure phenotypes over time (forebrain→brainstem seizures).

### C57BL substrains show varying seizure traits

#### Initial myoclonic jerk threshold

**Figure 1:**
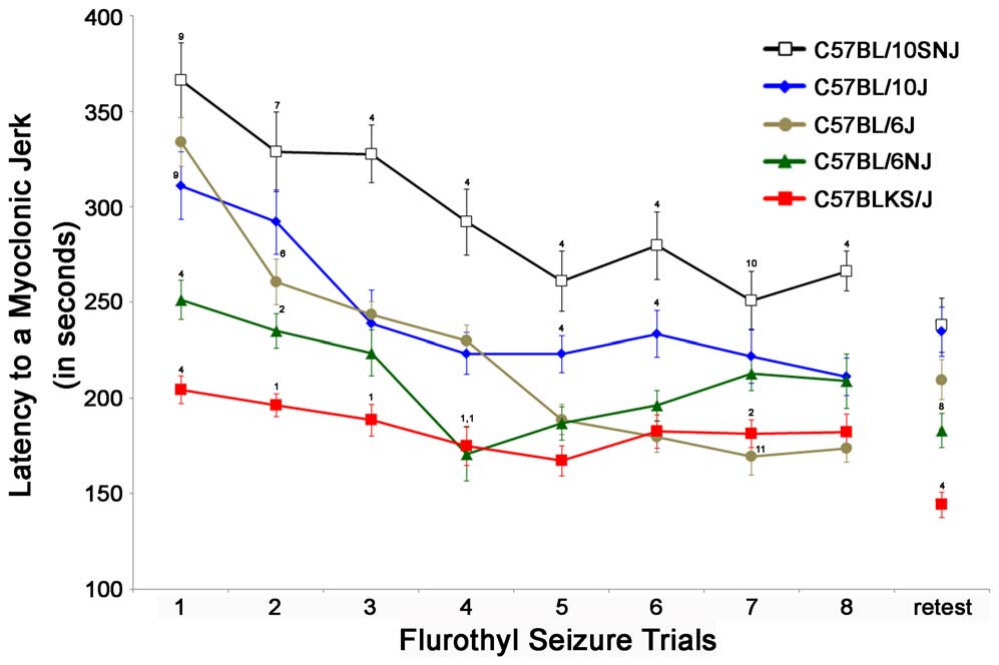
C57BL substrain differences in myoclonic jerk thresholds. The latency to the first myoclonic jerk (myoclonic jerk threshold (MJT)) on each seizure trial was determined for 5 C57BL substrains (n = 12 mice/substrain: 10SNJ, 10J, 6J, 6NJ, and KSJ) by exposure to 10% flurothyl during eight induction trials followed by a 28-day rest period and a single flurothyl retest. 10SNJ and 6J mice have the highest baseline MJT that are statistically indistinguishable. However, 6NJ and KSJ mice have significantly lower initial MJT as compared to all other substrains (*P*<0.0001). Additionally, four of the five individual substrains (6J, 10SNJ, 10J, 6NJ) showed significant differences in MJT across the 8 seizure trials (*P*<0.0001), except KSJ (*P* = 0.09). ^1^significantly different from 10SNJ, 10J, and 6J (*P*<0.02); ^2^significantly different from 10SNJ and 10J (*P*<0.04); ^4^significantly different from all other substrains (*P*≤0.05); ^6^significantly different from 10SNJ and KSJ (*P*<0.02); ^7^significantly different from 6J, 6NJ, and KSJ (*P*<0.01); ^8^significantly different from 10SNJ, 10J, and KSJ (*P*<0.02); ^9^significantly different from all substrains except 6J (*P*<0.03); ^10^significantly different from 6J and KSJ (*P*<0.04); ^11^significantly different from 10SNJ, 10J, and 6NJ (*P*<0.03).

Previously, we have shown that myoclonic jerk thresholds are significantly different between 6J mice and other inbred strains [29], so here we examined whether differences were observed between C57BL substrains. C57BL/10SNJ (10SNJ) and 6J mice have the highest baseline myoclonic jerk thresholds (MJTs) that are statistically indistinguishable (Fig. 1). However, C57BL/6NJ (6NJ) and C57BLKS/J (KSJ) mice have significantly lower initial MJTs as compared to all other substrains (Fig. 1; F_4,55_ = 20.76, *P*<0.0001).

#### Decreases in MJT over 8 seizure trials

Repeated measures ANOVA were used to determine whether each of the substrains showed significant differences in MJT across the 8 seizure trials. Decreases in MJT over 8 flurothyl-induced seizures were significantly different between the C57BL substrains (F_4,55_ = 38.15, *P*<0.00001), between trials (F_7,55_ = 43.35, *P*<0.0001), and as a strain by trial interaction (F_28,55_ = 3.88, *P*<0.0001) (Fig. 1). More specifically, four of the five individual substrains (6J, 10SNJ, 10J, 6NJ) showed significant differences in MJT across the 8 seizure trials (6J: F_7,77_ = 45.62, *P*<0.0001; 10SNJ: F_7,77_ = 8.26, *P*<0.00001; 10J: F_7,77_ = 9.93, *P*<0.00001; 6NJ: F_7,77_ = 5.78, *P*<0.00002). No differences were found in KSJ mice (F_7,77_ = 1.84, *P* = 0.09) (Fig. 1). Significant differences between substrains on each seizure trial are noted in Figure 1.

To determine whether the rate of decreases in MJT across the 8 seizure induction trials were different between the substrains, the slopes of the decreases in MJT across trials were calculated. There were significant differences between the slopes of the substrains (Fig. 1 and Table 1; F_4,55_ = 9.24, *P*<0.00001). 6NJ and KSJ mice had shallow slopes, 6J mice had the steepest slope with 10SNJ and 10J mice having comparatively moderate slopes (Fig. 1 and Table 1).

**Table 1:** The slopes of the myoclonic jerk thresholds and generalized seizure thresholds across the 8 trial flurothyl induction phase in C57BL substrains.

C57BL Substrain (n = 12/substrain)	Myoclonic Jerk Threshold (slope ± SEM)	Generalized Seizure Threshold (slope ± SEM)
C57BL/6J	−21.57±2.03	−28.69±1.57
C57BL/10SNJ	−15.06±2.71	−18.67±2.03
C57BL/10J	−12.70±3.24	−21.90±3.90
C57BL/6NJ	−5.64±2.51	−12.81±2.56
C57BLKS/J	−3.05±1.31	−10.84±1.68

Two of the five substrains (6J and KSJ) had significantly different MJT on the 28-day flurothyl retest as compared to their MJT on trial 8 of the induction phase (6J: t_12_ = 2.85; *P*<0.01; KSJ: t_12_ = 3.62; *P*<0.002; Fig. 1).

#### Initial generalized seizure threshold

**Figure 2:**
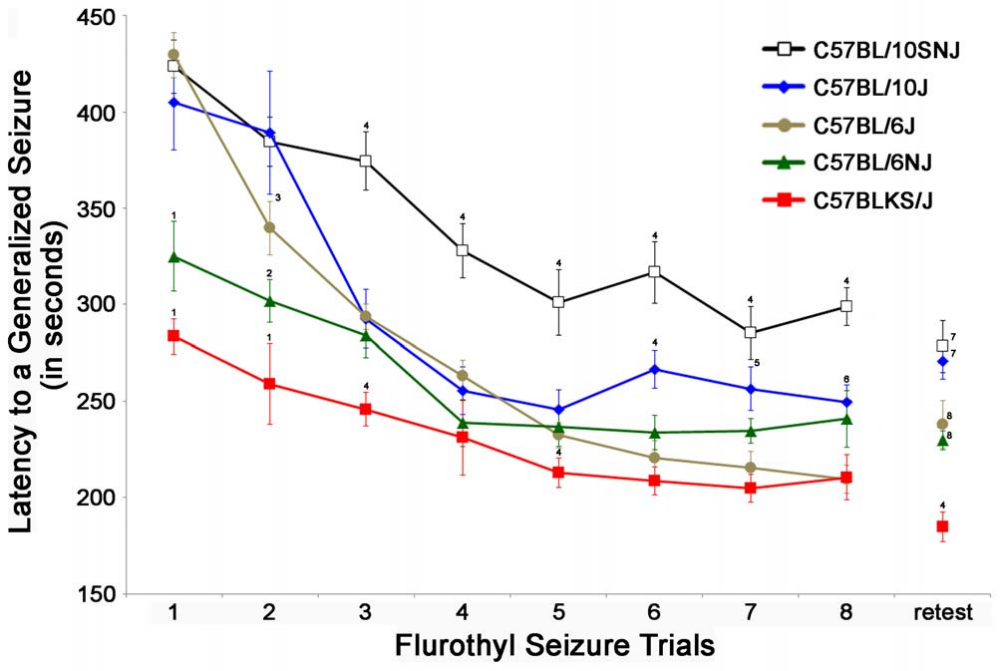
C57BL substrain differences in generalized seizure thresholds. The latency to a generalized seizure (generalized seizure threshold (GST)) on each seizure trial was determined for 5 C57BL substrains (n = 12 mice/substrain: 10SNJ, 10J, 6J, 6NJ, and KSJ) by exposure to 10% flurothyl during eight induction trials followed by a 28-day rest period and a single flurothyl retest. The baseline GST of 10SNJ mice and 10J mice were similar to that of 6J mice, whereas 6NJ and KSJ mice have significantly lower initial GST (*P*<0.01). For all C57BL substrains, there is a significant decrease in GST following repeated seizures (*P*<0.0001), which was independent of their initial GST. On flurothyl rechallenge, GST did not differ from their corresponding last seizure (seizure trial 8). ^1^significantly different from 10SNJ, 10J, and 6J (*P*<0.01); ^2^significantly different from 10SNJ and 10J (*P*<0.01); ^3^significantly different from KSJ (*P*<0.01); ^4^significantly different from all other substrains (*P*<0.05); ^5^significantly different from 10SNJ, 6J, and KSJ (*P*<0.05); ^6^significantly different from 10SNJ and KSJ (*P*<0.05); ^7^significantly different from 6J, 6NJ, and KSJ (*P*<0.05); ^8^significantly different from 10SNJ, 10J, and KSJ (*P*<0.05).

10SNJ and 10J mice have baseline GST that are statistically indistinguishable from 6J mice (Fig. 2). However, 6NJ and KSJ mice have significantly lower initial GST as compared to 10SNJ, 6J, and 10J mice (Fig. 2; F_4,55_ = 16.04, *P*<0.0001).

#### Decreases in GST over 8 seizure trials

Repeated measures ANOVA were used to determine whether each of the substrains had differences in GST across the 8 seizure induction trials. Decreases in GST over 8 flurothyl-induced seizures were significantly different between the C57BL substrains (F_4,55_ = 37.75, *P*<0.00001), between seizure trials (F_7,55_ = 89.87, *P*<0.0001), and as a strain by trial interaction (F_28,55_ = 3.77, *P*<0.0001) (Fig. 2). More specifically, each of the individual substrains showed significant differences in GST across the 8 seizure trials but plateaued at different seizure trials (6J: F_7,77_ = 74.49, *P*<0.00001 (plateaued on trial 5); 10SNJ: F_7,77_ = 16.98, *P*<0.00001 (plateaued on trial 4); 10J: F_7,77_ = 16.85, *P*<0.00001 (plateaued on trial 3); 6NJ: F_7,77_ = 8.51, *P*<0.00001 (plateaued on trial 4); KSJ: F_7,77_ = 11.29, *P*<0.00001 (plateaued on trial 3); Fig. 2). Significant differences between substrains on each seizure trial are highlighted in Figure 2.

To determine whether the rate of decreases in GST across the 8 seizure induction trials were different between the substrains, slopes of the GST decreases were calculated. With this analysis, there were significant differences between the slopes of the substrains (Fig. 2 and Table 1; F_4,55_ = 8.27, *P*<0.00001). Whereas 6NJ and KSJ mice had shallow slopes, 6J mice had the steepest slope with 10SNJ and 10J having comparatively moderate slopes (Fig. 2 and Table 1).

All of the substrains tested maintained their GST upon the 28-day flurothyl retest compared to the GST for trial 8 of the induction phase (no significant differences; Fig. 2).

#### Changes in seizure complexity over time

**Figure 3:**
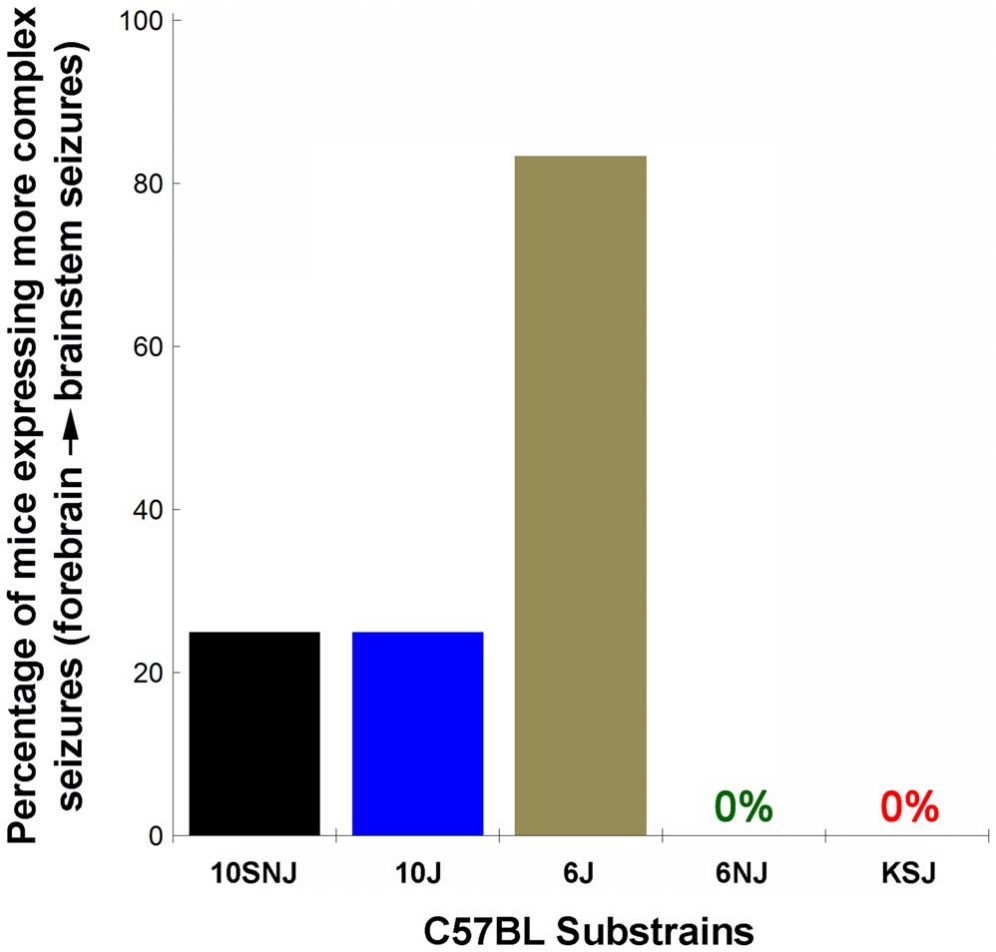
Flurothyl-induced seizure behaviors in C57BL substrains following 8 seizures, a 28-day incubation phase, and a final flurothyl challenge. While none of the 6NJ and KSJ mice expressed a more complex forebrain→brainstem seizure on flurothyl rechallenge, 25% of 10SNJ mice, 25% of 10J mice, and ∼80% of 6J mice did express a more complex forebrain→brainstem seizure. This demonstrates that the evolution of more complex seizures, following exposure to the repeated-flurothyl model, is controlled by alleles in the B6 genetic background. Chi-square analysis demonstrated a significant difference between substrains (Χ_4_ = 28.45; *P*<0.001; n = 12/substrain). Two out of 60 mice tested died on flurothyl retest (two B6 mice had a grade 7 seizure (tonic forelimb/hindlimb extension followed by death)).

In agreement with previous published results, ∼80% of 6J mice expressed a more complex forebrain→brainstem seizure on flurothyl retest (Fig. 3)[11], [12]. For the C57BL substrains, we found that 25% of 10SNJ mice (*P*<0.04) and 25% of 10J mice (*P*<0.04) expressed a forebrain→brainstem seizure phenotype (Fig. 3). In addition, none of the 6NJ and KSJ mice expressed a more complex seizure phenotype on flurothyl rechallenge (Fig. 3). Chi-square analysis demonstrated that there were significant differences between substrains (Χ_4_ = 28.45; *P*<0.001). Lastly, of the 60 mice in 5 substrains rechallenged with flurothyl following the incubation phase, two 6J mice had a grade 7 seizure (tonic forelimb/hindlimb extension followed by death).

### Heritability of these seizure traits among the C57BL substrains

With respect to initial MJT and changes in MJT over time, initial GST and changes in GST over time, and changes in seizure complexity, the mode of inheritance for each of these seizure traits was calculated and demonstrated to be under genetic control (MJT: *H^2^* = 0.60; MJT across 8 seizure trials: *H^2^* = 0.48; GST: *H^2^* = 0.54; GST across 8 seizure trials: *H^2^* = 0.43; forebrain→brainstem: *H^2^* = 0.47). It is notable that the substrains with steeper GST slopes (the rate at which GST decreases across the 8 seizure induction trials) had a greater likelihood for changing their seizure phenotype (r_pb_(58) = −0.26, *P*<0.05).

**Figure 4:**
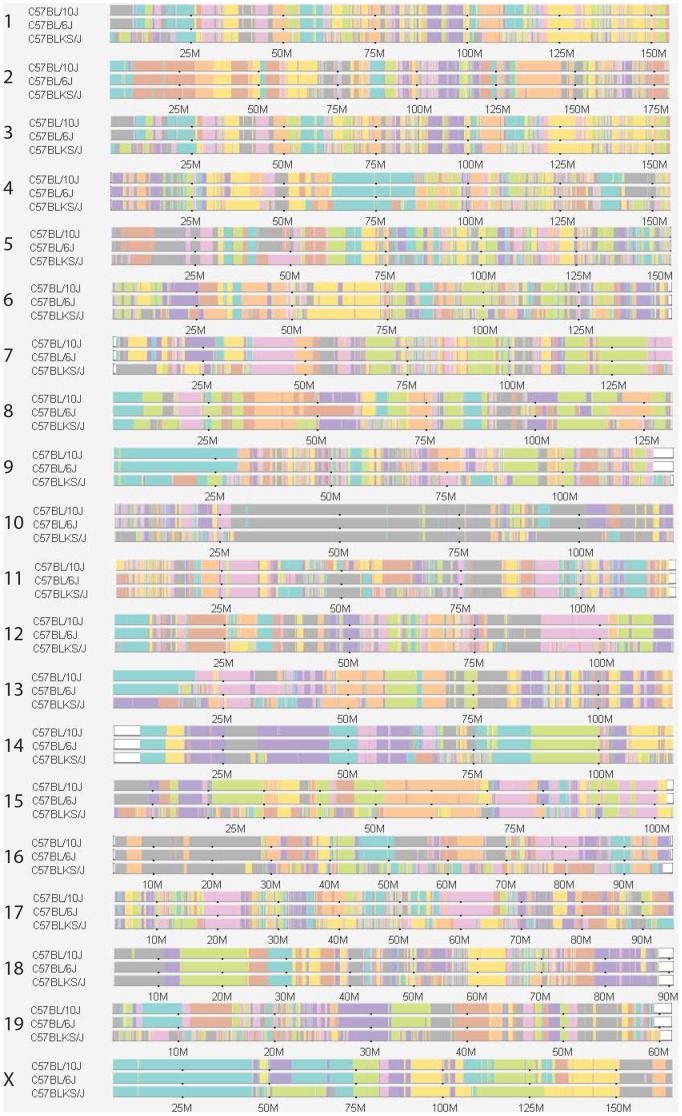
Illustration of haplotype diversity between 10J, 6J and KSJ mice. Each horizontal bar represents one of the mouse chromosome haplotypes. Differential shading between segments depict ancestral haplotype blocks consistent with the default parameters described on the mouse phylogeny viewer website. Differences in shading between lines show regions of genetic divergence between strains.

In an attempt to correlate the seizure phenotypic diversity within these substrains with existing genetic data, we queried the publically available data on the mouse phylogeny viewer (http://msub.csbio.unc.edu). Data were only available for 3 of the 5 strains studied here, but haplotypes from these strains indicate many regions that are similar along with numerous regions of haplotype diversity that are scattered throughout the genome (Fig. 4). Genetic diversity is greatest between the 6J and KSJ lines. Thus, any attempt to perform association studies will require a significant increase in the number of substrains studied, or an alternative mapping approach to localize our effects to a specific QTL region followed by a localized haplotype analysis.

## Discussion

Among the inbred strains evaluated to date in the repeated-flurothyl model, C57BL6/J (6J) mice have shown the most interesting responses in modeling: seizure progression through changes in GST following repeated seizures and the evolution of more complex seizures over time [11]. Our survey of five substrains of closely related C57BL mice demonstrates that genetic control over these seizure characteristics is divergent among their shared ancestry. Other studies have found similar significant differences between C57BL substrains in alcohol preference, pain threshold, fear conditioning, and maximal electroshock seizures that are in part attributable to genetic differences [3], [45]–[49] indicating that these sublines may be a fruitful source for haplotype refinement in positional cloning studies. Recently, cocaine responsivity in C57BL/6 and C57BL/6N substrains was mapped to a QTL responsible for 70% of this phenotypic difference leading to the identification of a nonsynonymous mutation in the cytoplasmic FMRP interacting protein 2 gene (*Cyfip2*) [50]. This study highlights the importance of examining mouse substrains as a powerful approach to reveal important genetic causes or modifiers of a phenotype or trait.

Since seizures are multifactorial, it is important to demonstrate whether specific effects on seizure susceptibility are a result of genetic influences or are related to the inherent properties of the convulsive stimuli. While no previous study has systematically analyzed these substrains with flurothyl, Ferraro et al., 2004 and 2011 demonstrated that, for baseline GST, C57BLKS mice are more susceptible to maximal electroconvulsive shock induced seizures compared to C57BL/10Sn, C57BL/6, and C57BL/10 mice. In fact, there was a trend for C57BL/10Sn mice to have higher maximal electroshock seizure thresholds than C57BL/6 mice, and for C57BL/6 mice to have higher thresholds than C57BL/10 mice [2], [3]. This was similar to what we observed with flurothyl, particularly following repeated exposures to flurothyl, indicating that these differences in seizure susceptibility are genetic and are not the result of the specific type of convulsant used.

Our data suggest that while a percentage of mice in 3 of the 5 substrains tested undergo a change in seizure phenotype to a forebrain→brainstem seizure following seizure induction, incubation and flurothyl retest (6J, C57BL/10SNJ (10SNJ), and C57BL/10J (10J) mice), these substrains also have comparable baseline GST, with the substrains not changing their seizure phenotype (C57BL/6NJ (6NJ) and C57BLKS/J (KSJ) mice) having significantly lower initial GST. This may indicate that strains of mice with higher GST may be more susceptible to developing more complex seizures over time. Indeed, we have previously reported that BALB/cJ, C3H/HeJ, and 129S1/SvImj mice have GST similar to 6J mice, and also have a high percentage of mice expressing forebrain→brainstem seizures during the flurothyl induction phase [11]. Understanding the pathophysiological processes underlying these plasticity changes could potentially lead to the development of new therapeutics directed against novel targets.

In addition to seizure complexity, we also observed substrain differences in the reductions in GST over the 8 seizure induction trials (“kindling”). Notably, while 6J, 10J and 10SNJ mice have comparable GST on the 1^st^ day of induction, 6J, 10J, and 6NJ mice are most similar by day eight. This indicates that although initial GST has a direct impact on the rate of kindling, independent processes that segregate within C57BL substrains also modify this phenotype. While it is impossible to formally test this without experimental crosses, these data support an additive model for seizure threshold, where 10SNJ, 10J and 6J mice retain more alleles contributing to high GST than 6NJ or KSJ mice. However, all of the substrains appear to “kindle” to some extent with their absolute rate depending on their initial GST.

Despite the close phylogenetic relationships between these lines, our initial attempts to correlate genotype with phenotype have shown that there is likely insufficient power for such an analysis on these data. Genetic divergence between these substrains is widespread particularly in KSJ, which not surprisingly shows the greatest difference in seizure progression from 6J. However, utilization of strains like 6NJ and 6J, in conjunction with alternative mapping approaches, should provide critical data to focus our attention on the causative mutations in future work.

In penicillin-induced epilepsy models, 6J mice showed differences in the relative power of delta, theta, alpha, beta, and gamma bands compared to BALB/c mice, that is partly due to the differences in their seizure susceptibility [7]. Similarly, EEG power spectrum analyses in C57BL substrains exposed to the repeated-flurothyl model may reveal important insights regarding these frequency bands. Interestingly, in electrical kindling, a recent report showed that the evolution of high frequency discharges, particularly in the beta and gamma frequency range, predicted the future appearance of fully kindled seizures in kindled rabbits [51]. Therefore, it would be particularly interesting to determine whether differences/changes in EEG frequency bands across the repeated-flurothyl model, in C57BL substrains, and especially in 6J mice, can predict whether a mouse will change its seizure phenotype upon flurothyl rechallenge. Identification of such an EEG signature could serve as an important biomarker that could predict whether an individual might develop more complex seizures over time.

Currently, we are taking advantage of the phenotypic diversity across all inbred strains to determine the major QTL controlling seizure traits in the repeated-flurothyl model. These data in conjunction with deep sequencing of the discovered QTL regions in C57BL substrains should help refine the candidate intervals and aide in the discovery of causative mutations.
